# Aseptic Osteonecrosis of the Femoral Head: The Effect of Corticosteroid Therapy and Long COVID Syndrome

**DOI:** 10.7759/cureus.54327

**Published:** 2024-02-16

**Authors:** Sohayb Darraz, Mokhtari Omar, Adnane Lachkar, Najib Abdeljaouad, Hicham Yacoubi

**Affiliations:** 1 Orthopedics Department B, Centre Hospitalier Universitaire (CHU) Mohammed VI Oujda, Oujda, MAR; 2 Faculty of Medicine and Pharmacy, Mohammed First University, Oujda, MAR; 3 Traumatology and Orthopedics Department B, Mohammed VI University Hospital, Oujda, MAR; 4 Department of Orthopedic Trauma, Mohammed VI University Hospital, Oujda, MAR; 5 Department of Traumatology and Orthopedics, Mohammed VI University Hospital, Faculty of Medicine and Pharmacy, Mohammed First University, Oujda, MAR; 6 Department of Traumatology and Orthopedics, Mohammed VI University Hospital, Oujda, MAR

**Keywords:** arthroplasty, corticoids, total hip replacement (thr), osteo-necrosis, sars-cov-2

## Abstract

SARS‑CoV‑2 infection remains a hot topic; it is characterized by its multi-systemic involvement. Corticosteroid intake has been the subject of worldwide attention as a potentially effective treatment against coronavirus disease 2019 (COVID-19). Corticosteroids are registered on the WHO list of essential medicines, easily accessible for a low price, and particularly useful for different categories of people.

The authors highlight the impact of corticosteroid administration for COVID-19 treatment on the occurrence of aseptic osteonecrosis in the femoral head. They also examine the pace of onset in comparison to corticosteroid usage unrelated to COVID-19.

This article presents a patient with osteonecrosis of the femoral head after taking corticosteroid therapy in the treatment of COVID-19. The dose taken by the patient is 90 mg of dexamethasone equivalent to 600 mg of prednisone.

The patient experienced the onset of OTA, and the duration of development was three months, indicating a relatively brief period. Comparison was made with data from the literature from 6 months to 1 year after taking corticosteroids in a context outside of COVID-19.

## Introduction

Aseptic osteonecrosis is reflected by ischemic necrosis of various cellular contingents. Aseptic osteonecrosis of the femoral head (AOF) presents the most frequent entity, resulting in circulation disturbance due to several factors. Corticosteroid therapy is the leading cause of nontraumatic AOF. It decreases femoral perfusion through mechanisms including vascular endothelial injury and microvascular thromboses [1].

In the acute phase of coronavirus disease 2019 (COVID-19), corticosteroids are used as lifesaving agents and are maintained for a longer period if the patient retains the known symptoms of COVID-19 (known as long COVID) [2].

## Case presentation

A 64-year-old individual presented with a history of hypertension on monotherapy for the past decade and hypertensive heart disease managed with DL-lysine acetylsalicylate and bisoprolol fumarate, as well as dyslipidemia treated with statin. Following a SARS-CoV-2 infection in 2021, the patient was put under corticosteroid therapy in the form of dexamethasone at a dosage of 6mg per day for a duration of 15 days. Noteworthy, there is no history of alcoholism or smoking.

The appearance of symptoms dates back seven months by the onset of progressive pain in the lumbar spine radiating toward the left lower limb of mechanical appearance, with functional impotence, without any tingling sensation, claudication, numbness, or inflammatory signs associated.

Worsening of the symptoms occurred four months ago by accentuation of the pain in the folds of the groin becoming disabling with an inflammatory appearance with functional repercussions, especially when walking slowly up the stairs.

The general examination found a conscious patient who was stable on both hemodynamic and respiratory levels, with a PMA (Postel-Merle d'Aubigné) score of 3.

Examination of walking and standing was possible but with a crutch. There was shortening of 1 cm of the left lower limb, and the morphotype was normally focused.

**Figure 1:**
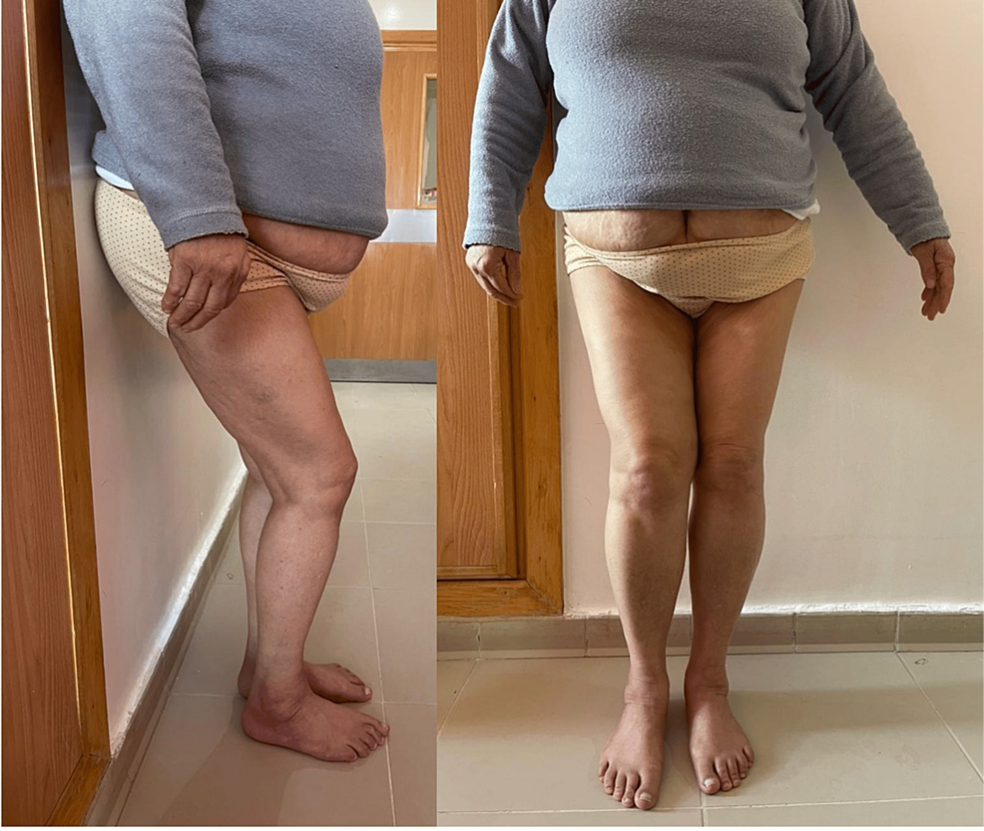
Clinical picture of the patient showing the crutch standing position with shortening and flessum of the knee.

The left hip joint displayed specific angles: flexion at 90°, extension at 10°, internal rotation at 15° with restricted external rotation, abduction at 10°, and adduction at 15°. An observed hip flexure of 10° during dorsal decubitus was noted. Additionally, muscle weakness in the gluteus medius was observed, along with the presence of a 10-degree knee flessum, which resolves when in a lateral decubitus position. Notably, these findings indicated limitations in the range of motion compared to normal hips, rendering activities such as squatting and cross-leg squatting impossible. Moreover, the patient faced difficulty in maintaining monopodal standing support. The rest of the clinical examination did not reveal any notable abnormalities (Figure 1).

**Figure 2:**
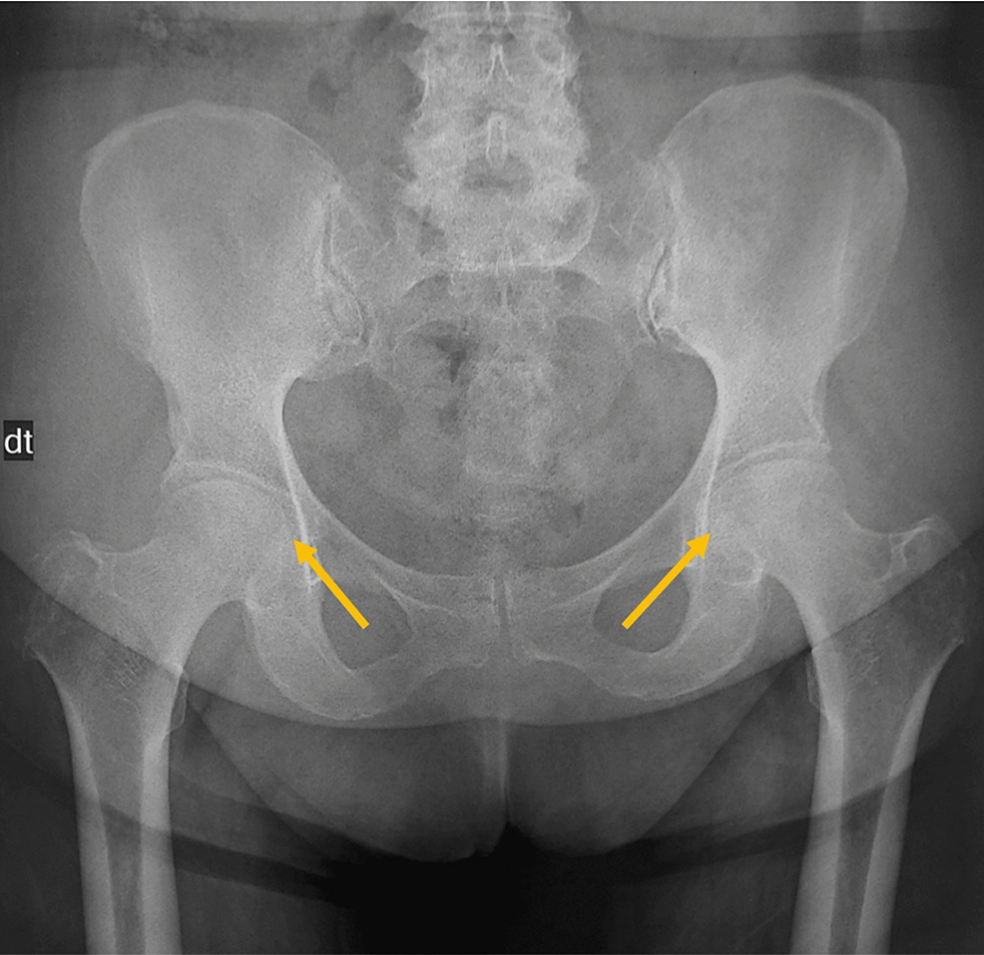
Standard radiography shows loss of sphericity of the femoral head.

**Figure 3:**
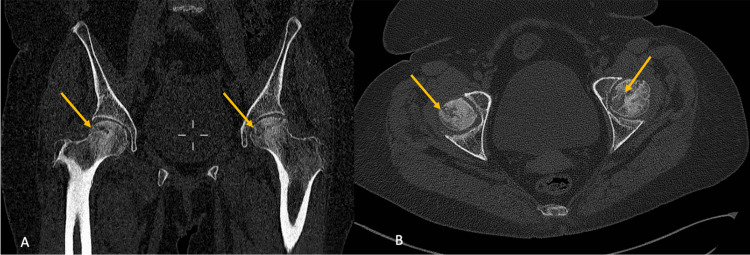
CT of the pelvis bone window: (A) Coronal section and (B) axial section show the alteration of the asterisk by loss of its rays and by peripheral increase of the density which translates into extensive necrosis of the femoral head stage 3 according to Ficat for the left femoral head and stage 2 for the right one.

Standard radiography shows loss of sphericity of the femoral head (Figure 2), and the computed tomography scan of the pelvis showed bilateral femoral osteonecrosis classified as stage 3 of Ficat classification on the left and stage 2 of Ficat on the right (Figure 3).

**Figure 4:**
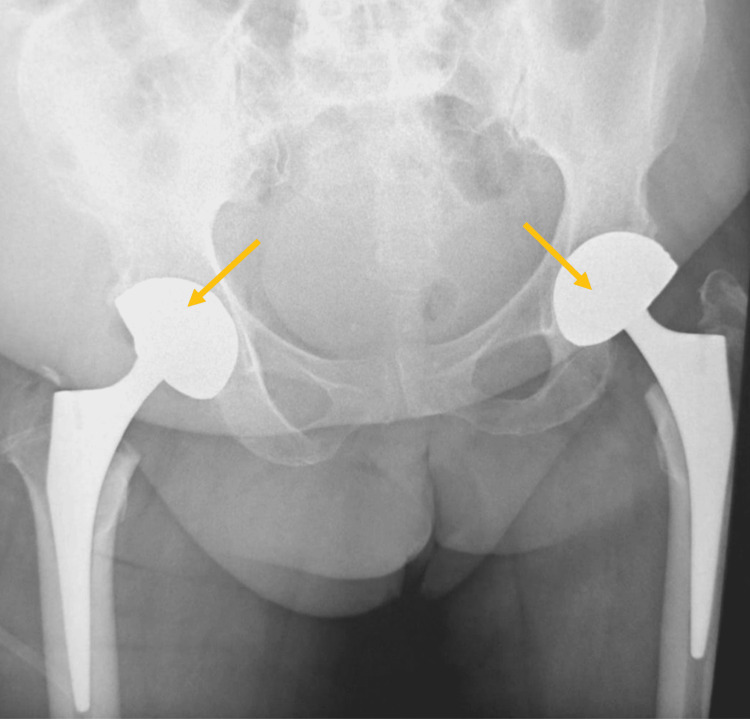
Postoperative plain radiography after a total hip replacement.

We also note that the patient underwent a standard biological assessment returning without particularity. From these clinical and radiological criteria, we have realized a cementless left total hip prosthesis and then a second prosthesis after four months (Figure 4).

 Two months after the second surgery, the patient underwent a period of rehabilitation and recovered her functional activity with possibly walking without help, absence of pain, and good progress in daily life.

## Discussion

Since its identification in Wuhan, COVID-19 has profoundly impacted individuals' lives and well-being. Globally, more than 183 million confirmed cases of COVID-19 have been documented, resulting in 3.97 million recorded deaths [3,4]. The term "long COVID," introduced by the World Health Organization (WHO), characterizes a range of persistent symptoms following acute infection with SARS-CoV-2. Similar to the acute phase, long COVID inflicts damage on various bodily systems, including the respiratory, cardiovascular, neurological, endocrine, urinary, and immune systems. It presents itself through a diverse array of symptoms such as fatigue, dyspnea, cardiac irregularities, cognitive and attention-related issues, sleep disruptions, post-traumatic stress disorder, muscle discomfort, concentration difficulties, and headaches- all reported manifestations of long COVID [5].

Osteonecrosis predominantly impacts the hips, with the knee being the second most commonly affected site. It is categorized into two main types: spontaneous osteonecrosis, primarily found in the knee and typically observed in the elderly population, and secondary osteonecrosis, also known as atraumatic osteonecrosis. The latter tends to affect younger individuals and is often linked to factors such as corticosteroid use, kidney disease, and hematologic disorders [6].

The consideration of osteonecrosis of the femoral head involves a comprehensive assessment of the patient's condition, necessitating additional investigations for a confirmed diagnosis. Diagnostic procedures include imaging techniques such as MRI or CT scans at all stages, along with plain radiography for detecting more advanced lesions [7].

Presently, investigations into the impact of COVID-19 on the advancement of existing chronic conditions and the emergence of new diseases in humans are highly pertinent. Corticosteroid usage is identified as a prevalent factor contributing to the onset of AOF. The pathogenesis of steroid-induced AOF is not fully elucidated, but the proposed mechanisms encompass fat emboli, fat hypertrophy, a hypercoagulable state, vascular endothelial dysfunction, and abnormalities in bone marrow stem cells [8].

There is a lack of definitive data regarding the duration of corticosteroid absorption and dosage, both of which substantially elevate the risk of osteonecrosis. Jones and Koopman suggest that a minimum total dose of 2000 mg of prednisolone is necessary for the onset of osteonecrosis [9]. However, contrasting viewpoints exist, with some researchers contending that a lower dose of prednisolone, specifically 700 mg, is adequate for the development of osteonecrosis [10].

McKee et al. demonstrated in their study of 15 patients with AOF that the mean steroid equivalent dose was 850 mg, ranging from 290 mg to 3300 mg [11]. In our case, the patient received a 150 mg dose of dexamethasone for 15 days, equivalent to 900 mg of prednisolone. This suggests that even low-dose corticosteroid usage might contribute to osteonecrosis development.

Anderton and Helm presented a clinical case of osteonecrosis of the humeral head emerging two years after dexamethasone treatment [10]. According to McKee et al., the average time from corticosteroid treatment to clinical manifestation of osteonecrosis is 16.6 months, with notable variability ranging from 6 to 33 months [11]. Literature reviews on osteonecrosis pathogenesis indicate occurrences within 6 to 12 months after hormone use [12,13]. In the sole publication on post-COVID-19 osteonecrosis, the reported mean onset time was 58 days (45-67 days) [14]. In our patient, the time from corticosteroid intake to symptom onset was seven months.

This suggests that the development of osteonecrosis after COVID-19 occurs significantly faster than after hormone therapy. It can be inferred that the presence of other factors related to COVID-19 infection accelerates osteonecrosis development.

## Conclusions

The use of corticosteroids is considered one of the most frequent causes of the development of aseptic osteonecrosis of the femoral head, our research with the results reported in the literature states that the association of SARS-CoV 2 infection accelerates the evolution of the pathology.
